# Draft Genome Sequence of *Bordetella trematum* Strain HR18

**DOI:** 10.1128/genomeA.01357-14

**Published:** 2015-01-08

**Authors:** Dong-Ho Chang, Tae-Eun Jin, Moon-Soo Rhee, Haeyoung Jeong, Seil Kim, Byoung-Chan Kim

**Affiliations:** a Korean Collection for Type Cultures, Biological Resource Center, Korea Research Institute of Bioscience and Biotechnology (KRIBB), Yuseong-Gu, Daejeon, Republic of Korea; b Korean Bioinformation Center, KRIBB, Yuseong-Gu, Daejeon, Republic of Korea; c Division of Metrology for Quality of Life, Center for Bioanalysis, Korea Research Institute of Standards and Science (KRISS), Yuseong-Gu, Daejeon, Republic of Korea

## Abstract

The genus *Bordetella* is reportedly a human or animal pathogen and environmental microbe. We report the draft genome sequence of *Bordetella trematum* strain HR18, which was isolated from the rumen of Korean native cattle (Hanwoo; *Bos taurus coreanae*). It is the first genome sequence of a *Bordetella* sp. isolated from the rumen of cattle.

## GENOME ANNOUNCEMENT

The genus *Bordetella* comprises Gram-negative, glucose-nonfermenting coccobacilli of the phylum *Proteobacteria* and currently consists of eight species with validly published names (http://www.bacterio.cict.fr/a/acinetobacter.html). Usually, *Bordetella* species infect the respiratory tracts of humans. Specifically, the type species of the genus *Bordetella*, *B. pertussis* has been studied extensively owing to its pathogenesis with regard to whooping cough in humans. It recently reemerged as a major public threat despite the wide availability of vaccines (1). However, *B. trematum* has never been found in the respiratory tracts of humans or animals. Instead, it was isolated from wounds and from ear infections in humans (2) and was later identified from a diabetic ulcer and issuing from bacteremia (3, 4). *B. trematum* is usually considered a nonpathogenic microbe, but it is opportunistic. In the course of investigating the microbial diversity of the rumens of Korean native cattle and isolating culturable microbes, the *B. trematum* strain HR18 was isolated from the rumen fluids of cannulated cattle. In order to gain insight into the potential pathogenicity associated with the isolate, the whole genome sequence of strain HR18 was analyzed. In this report, we present the draft genome sequence of *B. trematum* strain HR18. *Bordetella* sp. has never been isolated from the rumen of cattle. Therefore, this is the first draft genome sequence of the genus *Bordetella* sp. isolated from bovine rumen.

The HR18 genome was sequenced using an Illumina HiSeq 2000 system at the Genome Research Center of the Korea Research Institute of Bioscience and Bioengineering (KRIBB). A total of 14,222,221 paired-end reads (341.62-fold coverage) were obtained from the HiSeq 100-bp paired-end library and were preprocessed and *de-novo*-assembled using the CLC Genomics Workbench (CLC bio), version 7.5. The genome sequence was assembled into 21 scaffolds (23 contigs). The sizes of the largest and the *N*_50_ contigs were 542,107 and 398,472 bp, respectively. The open reading frames (ORFs) of the assembled genome were predicted and annotated using the Integrated Microbial Genomes—Expert Review (IMG-ER) (5), NCBI Clusters of Orthologous Groups (COG) (6), Pfam (7), and EzTaxon-e (8) databases and the rRNA genes and tRNA genes were identified by utilizing the RNAmmer 1.2 (9) and tRNAscan-SE 1.23 (10) tools, respectively. The draft genome of *B. trematum* strain HR18 was 4,204,757 bp. The G+C content was 65.8%, and the ORF numbered 3,882. The numbers of tRNA and rRNA, and the protein coding genes with functions were 58, 3, and 2,612, respectively. The genome is estimated to contain 3,292 coding sequences, with the majority of the sequences coding for amino acid transport and metabolism. Approximately 183 genes are predicted to be involved in membrane transport. Interestingly, two genes coding for cytolethal distending toxin (CDT) were found in the genome of *B. trematum* strain HR18. CDT is a heterotrimeric B-type genotoxin produced by certain gammaproteobacterial mucocutaneous bacterial pathogens (11). Thus far, no research has shown that betaproteobacteria, *Bordetella* species produce CDT. The potential pathogenicity of *B. trematum* strain HR18 against Korean native cattle requires further investigation.

### Nucleotide sequence accession number.

The draft genome sequence of *B. trematum* is available in DDBJ/EMBL/GenBank under the accession no. JPQP00000000.
